# Four-dimensional multi-particle tracking in living cells based on lifetime imaging

**DOI:** 10.1515/nanoph-2021-0681

**Published:** 2022-03-16

**Authors:** Danni Chen, Heng Li, Bin Yu, Junle Qu

**Affiliations:** Center for Biomedical Optics and Photonics (CBOP) & College of Physics and Optoelectronic Engineering, Key Laboratory of Optoelectronic Devices and Systems of Guangdong Province and Ministry of Education, Shenzhen University, Shenzhen, 518060, China; Tsinghua-Berkeley Shenzhen Institute (TBSI), Tsinghua University, Shenzhen, 518055, China

**Keywords:** fluorescence lifetime microscopy, multi-particle tracking, single particle tracking

## Abstract

Research on dynamic events in living cells, such as intracellular transportation, is important for understanding cell functions. As movements occur within cells, the microenvironment of the moving vesicles or biomacromolecules may affect the behavior of them. Herein, we propose a method of simultaneously monitoring changes in spatial positions and the local environment related to the fluorescence lifetime, i.e., four-dimensional (4D) multi-particle parallel-tracking in living cells. Based on double-helix point spread function (DH-PSF) microscopy and streak camera, the method combines three-dimensional (3D) localization methods and fluorescence lifetime imaging. By modifying the PSF of the system, the 3D positions and fluorescence lifetime information for several molecules within a depth of a few microns can be acquired simultaneously from a single snapshot. The feasibility of this method is verified by simulating the real-time tracking of a single particle with a given trajectory. In addition, a proof-of-concept 4D tracking system based on the DH-PSF and streak camera was built. The experimental results show that the 3D localization and lifetime precision are *σ*(*x*, *y*, *z*) = (26 nm, 35 nm, 53 nm) and *σ*(*τ*) = 103 ps, respectively, and the effective depth of field is approximately 4 μm. Finally, intracellular endocytosis in a living cell was observed using the system, which demonstrated the successful 4D tracking of two microspheres moving within an axial depth of 4 μm. This work opens a new perspective for research of dynamic processes, by providing information about the chemical (microenvironments) and physical (positions) changes of moving targets in living cells.

As the basic units of lifeforms, cells are constructed via numerous molecular mechanisms and complicated biochemical reaction networks [1, 2]. In cells, a variety of biomacromolecules and vesicles often need to be transported between specific areas of cells. It is essential to analyze and describe the dynamics of these intracellular transport processes for understanding the life processes and physical environment related to intracellular molecular transport [3, 4]. In addition to the specific functional organelles which enclose themselves with membranes, there are dozens of different types of intracellular bodies that are not membrane-bound that will change the environments of their surroundings on the concentration, temperature, pH or salt ion concentration [5], [6], [7], [8], [9]. Furthermore, in other aspects drug delivery processes are also dependent on the cell environment. Drugs or molecules carrying drugs move within the cell, acting on different functional positions, via complex transport networks. The discovery of complex dynamic processes in living cells can greatly promote our understanding of the cellular and molecular mechanisms [10], [11], [12]. Thanks to ongoing developments in advanced microscopy, the possibility of visualizing and resolving individual molecules in natural contexts such as living cells is being translated into a more quantitative and accurate description of the spatiotemporal dynamic processes controlling cell function [13]. However, to date, only the preliminary frameworks of the complex mechanisms governing these sophisticated transportation events in cells have been revealed. In order to understand the entire transportation event, more information should be provided for the interpretation of intracellular dynamic processes. Considering that the microenvironment may affect or even dictate the trajectories of intracellular transportation, it is necessary not only to locate and track moving targets, such as biomacromolecules and vesicles, but also to monitor the microenvironment along the movement path [14, 15]. This monitoring is hereafter referred to as four-dimensional (4D) tracking, with three dimensions describing the spatial localization of the target and one dimension reporting the microenvironment of the target. The combination of biology and physical technology presents the opportunity for us to obtain more comprehensive models, allowing us to acquire a deeper understanding of molecular transport behavior in cells and environmental responses, as well as further revealing the interactions between many complex molecules and illuminating the physical mechanisms governing living cells [16].

For the purpose of localizing and tracking moving molecules and vesicles, single particle tracking (SPT) is the most commonly used tool, and this has been widely used in various cell biology fields, including membrane dynamics [17], kinesin dynamics [18], and gene regulation [19]. By analyzing signals from single particles, their positions can be determined with few-nanometer precision [13], and by the real-time imaging of these particles, their positions can be tracked over time and space. In early SPT experiments, particles were tracked by means of wide field imaging with charge coupled devices, and thus they could only be tracked in two dimensions. Tracking the movements of single particles in two spatial dimensions has proved to be a powerful method to explore the functions of biological molecules. However, strictly speaking, targets always move in three dimensions, and thus for most events, essentially for those that occur inside cells, such as protein and RNA trafficking, three-dimensional localization and tracking is important [20, 21]. In recent years, with the development of super resolution fluorescence microscopies, which allow the possibility of superseding the diffraction limit, a variety of 3D nano resolution positioning methods have emerged, such as those based on astigmatism, point spread function reconstruction, bifocal planes, etc. [22], [23], [24], [25]. Among these approaches, the double-helix point spread function (DH-PSF) method has several obvious advantages. It has a high Fisher information content in all three dimensions that remains relatively constant over a 2 μm depth of field (DOF). This means that the position precision obtained from DH-PSF is independent of the positions of the fluorescent molecules. In addition, the shape of the DH-PSF is basically undispersed within the entire DOF [26].

Despite the rapid development of SPT, SPT technologies are now mainly focused on the use of intensity information to obtain accurate three-dimensional motion trajectories, and information about changes in the microenvironment along the trajectories is not acquired. Thus, 4D-tracking cannot be achieved by means of existing SPT approaches. The fluorescence lifetime is an ideal candidate measurement parameter for microenvironment monitoring as it reflects the local microenvironment of the particle during motion [27, 28]. The fluorescence lifetime, which can also be expressed as the fluorescence index decay rate, is defined as the time required for the fluorescence emission to decay to 1/*e* of its initial intensity following excitation. It can be used to monitor the microenvironment because the fluorescence emission is related to the energy transfer between an excited molecule and its surrounding environment. Fluorescence lifetimes are very little affected by interference resulting from fluctuations in probe concentrations, changes in light exposure, and inhomogeneities in the optical properties of the medium, and thus these can provide more accurate detection results than fluorescence intensities. Fluorescence lifetime imaging microscopy (FLIM) has shown great potential for the development of biomedicine [29]. It has been used to discover the interaction mechanisms of drug targeting in different stages of cells [30]. In addition, FLIM has been used to study the dynamic processes and mechanistic changes for drugs in different cell-level microenvironments [30], [31], [32]. At present, there are several available technologies for measuring fluorescence lifetimes, including time-dependent single photon counting (TCSPC) [33], time-gating [34], and the use of streak cameras [35]. TCSPC is a statistical method, which means that for each measurement, only a single photon is detected; thus, to accumulate a sufficient number of photon events for the required statistical data precision, a specific dwell time is for each pixel must be used. Furthermore, the scanning mode used in TCSPC also requires time, and hence this method is not suitable for the dynamic imaging of living cells. In order to detect changes in the fluorescence lifetimes of moving molecules in living cells, real-time monitoring methods, such as the use of a streak camera, are preferable [36, 37]. As an ultrahigh-speed detector, the streak camera is an ideal detector for measuring fluorescence lifetimes by capturing the process of fluorescence decay. As a wide-field imaging technology, the streak camera method collects information on all the molecules in the field of view simultaneously, in a single-shot measurement.

In this paper, we propose a 4D-tracking method by combining the DH-PSF approach with the use of a streak camera. Thus, molecules in the field of view can be localized and tracked with nano-resolution in three dimensions. Furthermore, the fluorescence lifetimes of these molecules can also be acquired. A feasibility analysis is performed for the method via simulation. Numerical results show that particles existing within a depth of 4 μm can be tracked simultaneously, with nano-scale spatial resolution and a fluorescence lifetime precision of 40 ps. We then built a proof-of-concept 4D-tracking system, based on a home-built streak camera and a DH-PSF three-dimensional positioning microscope [38, 39], and observed endocytosis in a living RAW cell.

In summary, the proposed 4D-traking method is based on the stretching the traditional DH-PSF in one dimension so that the fluorescence lifetime information can be contained in this dimension.

As is described in previously published papers, the DH-PSF is formed via a so-called self-imaging effect by the superposition of a few Laguerre–Gauss (LG) modes located along a straight line in the LG modal plane [22]. Therefore, the DH-PSF presents invariant features that continuously rotate with the defocus over several microns, as shown in Figure 1. The DH-PSF consists of two lobes, and the transverse and axial positions of the emitter can be determined from the center and orientation angle of these two lobes. In our design, the DH-PSF is generated with a specifically designed phase pattern (up-right subfigure in Figure 1(a)). The phase mask can be implemented using a spatial light modulator (SLM) or can be fabricated. The phase mask is mounted at the Fourier plane in a 4*f* relay system (shown in Figure 1(a)), as per the strategy adopted in conventional DH-PSF microscopy. In addition, as in DH-PSF microscopy, a calibration curve of orientation angle versus axial position can be measured (Figure 1(b)).

**Figure 1:**
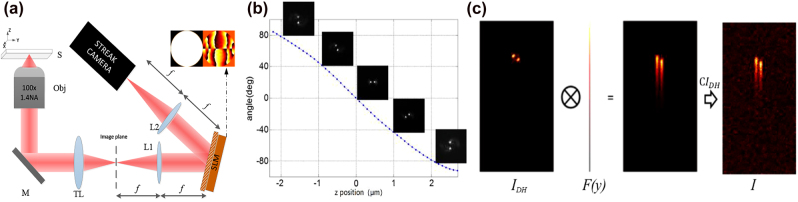
Principle of 4D-tracking method. (a) Collection light path of the 4D-tracking setup: the sample fluorescence of (S) is collected and imaged via an objective (Obj) and a tube lens (TL), and then modulated with a 4f relay system, consisting of two focal-length-matched achromatic lenses (L1 and L2) and a phase pattern implemented using an SLM, to generate the DH-PSF. (b) Calibration curve illustrating the relationship between the rotation angle of the two lobes of the DH-PSF and the z -axis position. (c) Schematic illustration of the convolution of the DH-PSF and a monoexponential function to generate the image acquired by the streak camera.

Encoding fluorescence lifetimes into the DH-PSF is accomplished by using a camera with high temporal resolution. Compared with traditional DH-PSF microscopes, in our design, a streak camera is used as the detector instead of an EMCCD, and a ps-pulsed laser rather than a CW laser acts as the light source. For an emitter with fluorescence lifetime τ , the fluorescence intensity undergoes an exponential decay, i.e., $F\left(t\right)=F_{0}\text{exp}\left(-t/\tau \right)$ , where $F_{0}$ is the initial fluorescence intensity after excitation. In streak images, one spatial dimension (e.g. the y axis) is assigned as the time axis, and a linear relationship between the position on the axis and the time corresponding to that position is utilized such that the fluorescence intensity decay with time is presented as the intensity decay with position along the y axis, i.e., $F\left(y\right)=F_{0}\text{exp}\left(-y/y_{0}\right)$ . Here, $y_{0}$ is the distance corresponding to the duration τ proportionally,

(1) $$y_{0}=\tau /d$$

where d is the sweep speed in fs/mm. In our design, it is a DH-PSF that is projected onto the cathode of the streak camera, so the streak image is actually the convolution of $I_{\text{DH}}$ and F(y) , where *I*_DH_ is the DH-PSF, as shown in Figure 1(c).

(2) $$I=I_{\text{DH}}⊗F\left(y\right)+CI_{\text{DH}}$$

where ⊗ indicates a convolution and C is the scattering coefficient. The scattering time of samples is very short relative to the fluorescence lifetime. Thus, the streak image contains not only information about the three-dimensional position but also on the fluorescence lifetime, i.e., it possesses 4D information on the emitter.

The streak image is processed as follows. First, the raw image is denoised, then candidates for further processing are screened out. Images of individual candidates are then surface-fitted by means of the nonlinear least square method. The fitting results consist of the positions of the two lobes, $\left(x_{1},y_{1}\right)$ and $\left(x_{2},y_{2}\right)$ , and the intensity decay length, $y_{0}$ . The transversal coordinates of the emitter in the local image are $\left(\left(x_{1}+x_{2}\right)/2,\left(y_{1}+y_{2}\right)/2\right)$ , while the axial position of the emitter is acquired according to the angle calculated using $\theta =\tan^{-1}[\left(y_{2}-y_{1}\right)/\left(x_{2}-x_{1}\right)]$ and the calibration curve. The fluorescence lifetime of the emitter can be calculated using Eq. (1). It should be noted that, although the streak camera can collect information of several emitters within a certain depth simultaneously, these emitters should be distributed sparsely. When the images of two emitters are overlapped in y -axis, multi-exponential instead of single-exponential fitting should be adopted.

In the simulations, the samples are assumed to experience widefield illumination by a pulsed laser. As shown in Figure 1, fluorescence with wavelength of 550 nm and lifetime of 2 ns is collected via an objective (NA = 1.4, 100×) and 4*f* relay system. A phase mask is mounted at the Fourier plane to generate a DH-PSF that is a superposition of LG modes (0, 0), (1, 3), (2, 5), and (3, 7). The effective axial range is set to 4 μm. Then, the modulated fluorescence is detected by a streak camera. The ratio of distance to time along the sweeping direction ( y axis) is 38 ps/pixel. The effective pixel size of the obtained streak images is 0.08 μm. It should be noted that the effect of pulse duration on the streak image is ignored because it is of the order of picoseconds, i.e., much shorter than the nanosecond fluorescence lifetime.

The numerical simulations consisted of several steps. First, as in conventional DH-PSF, a calibration curve of orientation angle versus axial position was simulated (Figure 1(b)). This ensures that the depth range for three-dimensional localization is between −2 and 2 μm. Then, the localization and lifetime measurement accuracies for the 4D tracking method were simulated. Since the DH-PSF is stretched along the y -axis, localization precision might be affected, and this effect might be different at different *z* positions. Therefore, images of a single fluorescent molecule at seven axial positions with a 0.4 μm interval between them were simulated. For reasons of practicality, all the images shown here were processed by applying Poisson noise and adding Gaussian noise. The total number of fluorescence photons collected was set to 8000, and the expected value and variance of the Gaussian noise were set to 0 and 0.01 respectively. As adopted in other methods, we defined the localization precision at a certain depth as the standard deviation of 100 estimated positions of a single molecule at that depth. For comparison, the localization precision of the conventional DH-PSF method is also presented. As is shown in Figure 2(a), the average localization precisions were found to be 10, 15, and 33 nm in x , y , and z directions, respectively, and the precision of the fluorescence lifetime was 40 ps. The localization accuracies of the conventional DH-PSF and 4D tracking methods in the x and y directions are both relatively constant over the entire depth range. The localization precision of the 4D tracking method in the x direction is even slightly better than that of the conventional DH-PSF method. However, for the 4D tracking method, because the y axis is also the time axis, the accuracies in direction of this axis and the z axis were found to be worse than those of conventional DH-PSF microscopy. The z precision is the worst at z=0 μm. Two main reasons account for this consequence. Because the two main lobes at z=0 μm are closest, the z precision there is assumed the worst.The second reason is the worsen y precision. Worse y precision will lead to worse z precision, and such influence becomes the worst when the angle of the two main lobes is $0^{{}^{\circ}}$ which means z=0 μm. The lifetime precision is constant at depths near z=0 μm, but becomes a little worse at depths near z=±2 μm. This is because, at these depths, the two lobes of the DH-PSF are almost aligned along the y axis, and the images of the two lobes overlap on the streak camera, as shown in Figure 2(b) (I) and (VI).

**Figure 2:**
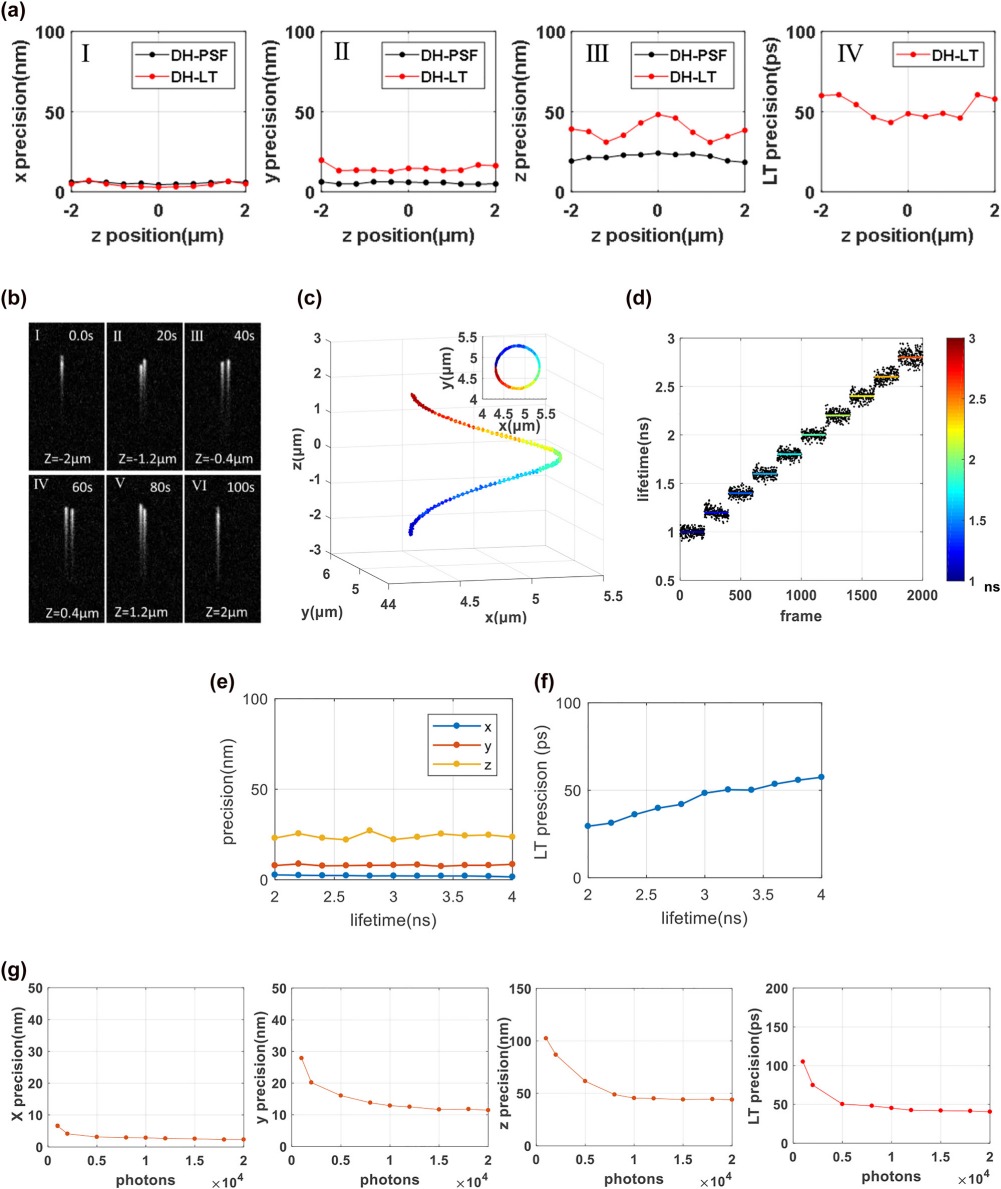
Numerical simulations of the 4D-tracking method. (a) Localization precisions in the x , y , and z directions (I)–(III) and accuracy of the fluorescence lifetime measurements (IV) analyzed statistically at different z positions (DH-LT, red dots). For comparison, localization precisions of conventional DH-PSF obtained with the same total number of photons are also presented for the same z positions (DH-PSF, black dots in (I)–(III). (b) Six images at six timepoints tracking the movement of a fluorescent emitter following a spiral trajectory and experiencing step-wise increases in fluorescence lifetime. It is apparent that sometimes the two lobes are separated (II)–(V) and sometimes they are overlapped (I), (VI). (c) Estimated localizations at timepoints are marked as dots with pseudo colors which reflect the corresponding fluorescence lifetime. (d) Ten fluorescence lifetimes at ten segments of the trajectory are analyzed further. (e) Spatial precision plotted against lifetime. (f) Lifetime precision plotted against lifetime. (g) Localization and lifetime precision at *z* = 0 μm, as a function of the number of total photons ranging from 1000–20,000 photons.

Next, the 4D-tracking method was applied to tracking an emitter originating at (*x*, *y*) = (54, 60) pixels and *z* = 2 μm, moving along a given trajectory: (400 sin(2*πt*/10), 400 sin (2*πt*/10), −40*t*) nm. Its fluorescence lifetime varied as (1 + ceil (*t*/10)*0.2) ns; thus, during the trajectory, the fluorescence lifetime of the emitter increased by 200 ps every 10 ms. As shown in Figure 2(c), the estimated positions and fluorescence lifetimes are in good agreement with those obtained via simulation. Next, the fluorescence lifetime data were extracted for further analysis. As shown in Figure 2(d), the average step size of 200.4 ± 1.6 ps for the 10 steps shown here is in good agreement with the step set in the 200 ps simulation. Furthermore, influence of the length of lifetime on the spatial precision and lifetime precision is also analyzed and shown in Figure 2(e) and (f), repectively. Although adding a temporal measurement decreased y and z precision, there is no significant correlation between the length of lifetime and the spatial precision, as is shown in Figure 2(e). However, as is shown in Figure 2(f), the lifetime precision has strong correlation with the length of lifetime, i.e., the longer the lifetime is, the worse the lifetime precision will be. It should be noted that we chose just one *z* position, *z* = −0.4 μm, for the analysis here. Finally, we analyzed the precisions at 1000, 2000, 5000, 8000, 10,000, 12,000, 15,000, 18,000, and 20,000 photons. As is shown in Figure 2(g), the more photons, the higher precisions. However, for a single specific fluorophore, more photons mean longer exposure time. So, in practice, there is a balance between the precision and temporal resolution. Using fluorophores with higher quantum efficiency or using microspheres containing more fluorophores are possible solutions. However, it should be noted that using single fluorophores as lifetime indicators here is indeed more difficult due to the low signal flux and troubles of photobleaching/blinking. This is one restriction of the method proposed here.

Further, the 4D-tracking method was first tested by moving 100 nm microspheres, and then demonstrated by the observation of endocytosis in a living RAW cell. The 100 nm carboxylate-modified microsphere solution was purchased from Thermo Fisher Scientific. The peak emission wavelength of the microspheres is 550 nm. All chemicals were of analytical grade. The solutions were first diluted with pure water to obtain the desired concentrations. Then, the diluted solutions were sonicated for 5 min at room temperature (25 °C). After sonication, 6 μL of the microsphere solution was pipetted onto a coverslip, and the microspheres were fixed to the coverslip surface. The fluorescent microspheres were then mixed with 0.5% low-melting agarose and then spread onto a glass slide.

A schematic of the 4D-tracking system is shown in Figure 3(a). A home-made ps-pulsed laser of 514 nm with a repetition rate of 1 MHz and pulse width of 10 ps was used as the excitation light source. After passing through a filter (EX Filter, ZET514/10x), the beam was expanded and collimated using a lens pair (beam expansion), and then introduced into a commercial microscope (IX81, Olympus). Samples were excited using a widefield mode through a tube lens (TL1) and an oil immersion objective (NA 1.4, 100×, Olympus). The fluorescence signal from the samples was collected by the same objective before being separated from the excitation beam by a dichromatic mirror (DM, ET525lp) and filter (EM Filter, FF01-559/34). After passing through a tube lens (TL2, *f*_tube_ = 180 mm), the fluorescence was modulated using a 4*f* system (L1 and L2), in which a DH-PSF phase mask (PM) is mounted at the Fourier plane. The DH-PSF mask was designed based on the theoretical analysis and numerical simulation reported previously [22], and it was fabricated on a fused quartz substrate via ion beam etching. Its clear diameter was approximately 5 mm, and the effective localization depth was designed to be 4 μm. The fluorescence was finally detected with a home-made streak camera. The streak camera was designed with a special slope voltage scanning circuit. It can work at a repetition frequency of ≤1 MHz. In order to detect multiple molecules simultaneously, the slit before the cathode of the streak camera was removed. To exploit the time resolution to its full extent, the streak camera and the laser pulses were synchronized by connecting the standard electrical sync output of the laser to the streak camera with a delay unit (Delayer). The trigger jitter of the scanning circuit was low, and the maximum time resolution of the camera was 10 ps [39]. The camera can be operated in static (imaging) or dynamic (sweep) mode, to acquire images not including or including fluorescence lifetime information, respectively.

**Figure 3:**
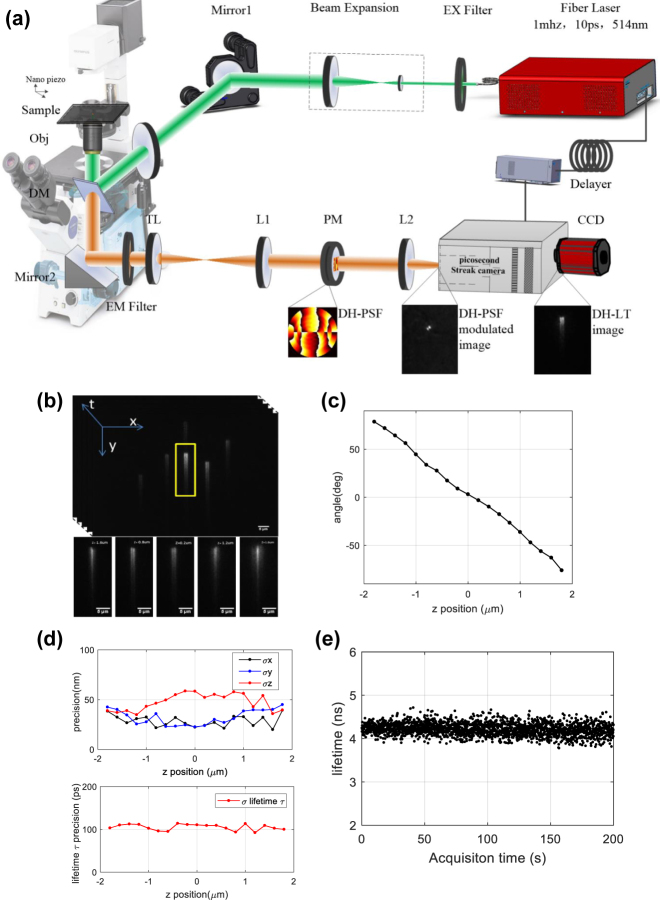
Experimental system for 4D-tracking and parameter testing results. (a) Schematic of the 4D-tracking system. The laser is expanded and collimated (Beam expansion) after an excitation filter (EX Filter), before, via an objective (Obj) combined with a tube lens (TL1) whose focal length is f = 180 mm, exciting the samples. The fluorescence signal is collected by the same objective and separated from the laser beam by a dichromatic mirror (DM). A 4f relay system consisting of two achromatic lenses (L1 and L2, f = 200 mm) and a DH-PSF phase mask (PM) mounted at the Fourier plane is positioned before the streak camera detector. (b) An image of a single microsphere is selected for parameter testing. The movement of the microsphere is controlled by a nano-piezoelectric stage, so the microsphere moves in the axial dimension in 100 nm steps along the z-axis. (c) Experimental calibration curve of the angles of the two lobes vs. axial position. (d) Localization precisions in the x, y, and z directions, shown as dark, blue, and red filled circles, respectively (upper plot), and precision of fluorescence lifetime measurements (lower plot) at different axial positions. (e) Successive measurements of the fluorescence lifetime in 200 seconds.

Considering that the actual lobe rotation angle at a certain defocusing position may be different from the theoretical one, as illustrated in Figure 1(b), before data acquisition, calibration is necessary. Single fluorescent microspheres were used as targets for calibration. The sample was mounted on a piezoelectric stage which was set to move along the z direction in steps of 200 nm. The motion of the stage and the exposure of the camera were synchronized to ensure the stage was stable throughout each exposure. An anti-axial-drift device with accuracy of less than 10 nm was also used here [40] (not shown in Figure 3(a)). A single bead in the sample was imaged and analyzed, as is shown in Figure 3(b). Images of the bead at five different depths are also shown as examples. The angles of the two lobes were then measured based on these images to generate the plot of angle versus axial position shown in Figure 3(c). The angle changed from −76.05° to 75.56° as the bead moved from −1.67 μm to 1.69 μm. To test the 4D-localization precision of the system, 100 images of the bead at each axial position were acquired consecutively, and data from 19 axial positions were analyzed. The interval time between two axial positions was set to 2 min which was long enough for stablizing the piezoelectric stage. Localization precision was determined as the standard deviation of the coordinates of the 100 position measurements. The exposure time was set to 40 ms. The average localization precisions in the x , y , and z directions were calculated from the corresponding localization precisions at all the positions shown in Figure 3(d): *σ*_*x*_, *σ*_*y*_, and *σ*_*z*_ were obtained as 26, 35, and 53 nm, respectively. Moreover, the lifetime precision of each axial position was also calculated to provide an average lifetime precision value *σ*_*τ*_ of 103 ps. The lifetime precision measured experimentally is worse than that estimated in Figure 2(f). Some reasons, such as non-ideal PSF because of the optical aberration, less photons, and higher noise level, may account for this. Next, the stability test was performed. The lifetime of fluorescent microspheres in a pH buffer (@37 °C and pH 7.4) was measured, by setting the detector to the mode of successive exposure with exposure time of 40 ms, which were the same settings for the following cell imaging. The fluorescence lifetime of a microsphere was measured successively in 200 s, and the lifetime measured at each time point was represented as a spot in Figure 3(e) shown below. The raw data was also attached in Supplementary materails as video 1. The mean of these measurements is 4.22 ns, with a standard derivation of 0.14 ns. The lifetime did not change significantly during 200 s.

Next, the 4D tracking system was used to observe the phagocytic pathway of fluorescent microspheres in living RAW 264.7 cells. RAW 264.7 cells are one type of immune cells. As immune regulatory effector cells, RAW cells kill microorganisms by phagocytosis. When foreign pathogens or foreign bodies are identified, the surface of the RAW cell becomes invaginated before they are then ingested by the cell. In these experiments, 100 nm fluorescent microspheres were used as model foreign bodies, and their positions and corresponding fluorescence lifetimes were monitored during the phagocytic process.

For the localization of microspheres inside living cells, RAW cells were cultured on 35 mm glass Petri dishes. Minimum essential cell culture medium with 10% fetal bovine serum supplement (1.5 mL) was added to the dish. The cell culture was incubated at 37 °C under 5% CO_2_. After 70% confluency was achieved, the dish was rinsed with 10 mM phosphate buffered saline (PBS) at pH 7.4, and then the cells were fixed with 4% paraldehyde in the same PBS buffer. After that, 30 μL of the microsphere solution was added to the Petri dish, and at the same time, data acquisition was commenced.

Considered that the environmental temperature might be a cause of the lifetime variation, during the cell experiment, the temperature of the sample was kept at 37 °C with a live-cell temperature controller (CU-501, LCI). During the stability test above, the sample was also kept in the temperature controller with the same settings. As was explained above, the lifetime in the stability test is 4.22 ± 0.14 ns, we believe that with the temperature controller, the temperature fluctuation is quite small and should not cause significant changes of the lifetime.

We analyzed two trajectories of single particles in the cells at different stages of endocytosis. The raw data was also attached in Supplementary materails as video 2. As is shown in Figure 4, the trajectories marked I and II are projected onto a wide-field image of the investigated cell cross-section, acquired under cell region in bright field mode (outlined in yellow, Figure 4(a)). For the two particles corresponding to the two traces, their fluorescence lifetime changed with time, as shown in Figure 4(b). Enlarged 3D plots of the two 4D trajectories are shown in Figure 4(c). The precise position of each location can be read out from the 3D coordinates, while the fluorescence lifetime at each position is also presented using a pseudo color scale. The change of fluorescence lifetime indicates that the particle may be subjected to different local environments during endocytosis (trace I) and the subsequent diffusion process (trace II).

**Figure 4:**
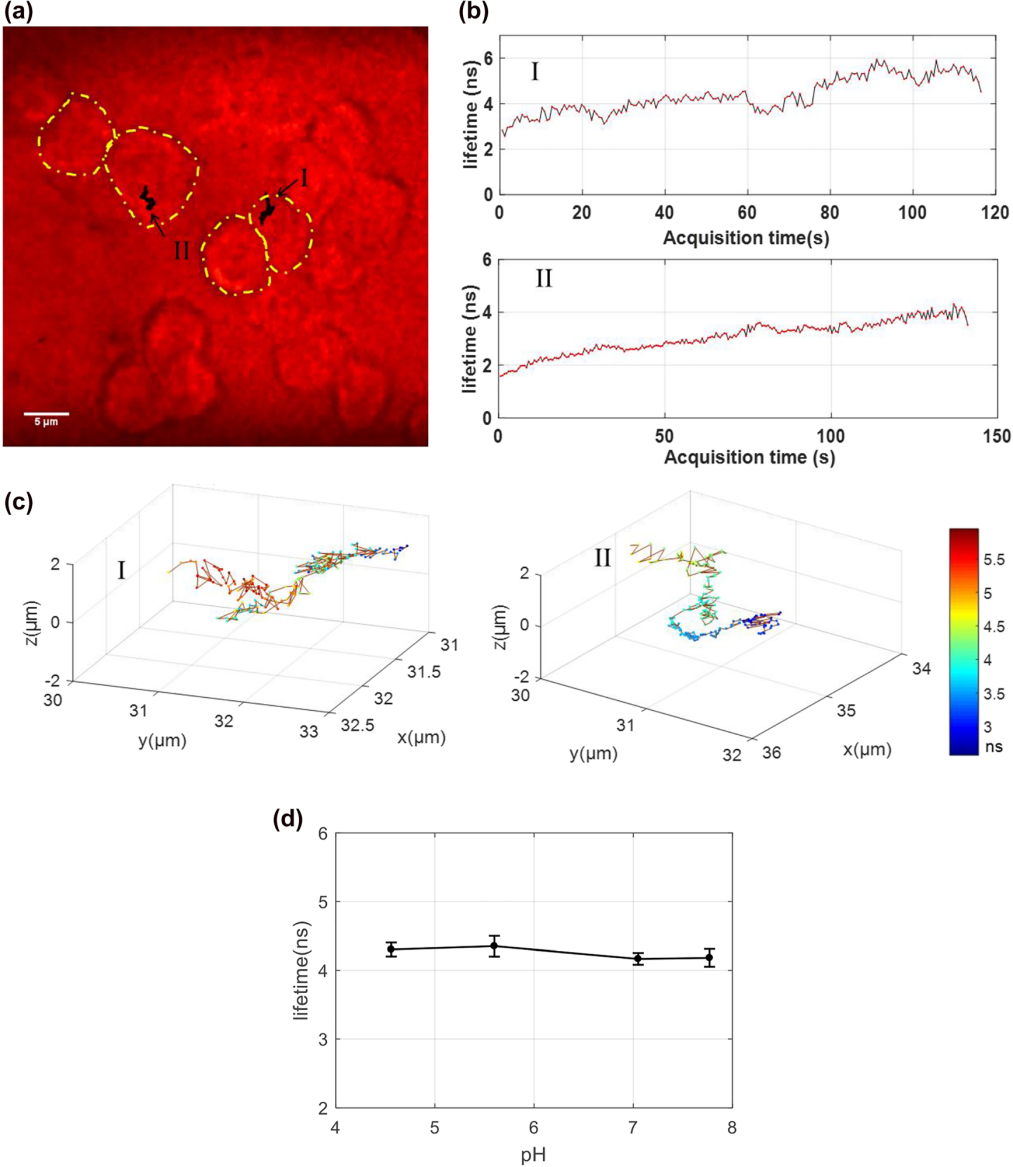
4D tracking of particles during endocytosis in living RAW cells. (a) To demonstrate the localization of the particles within the cell, trajectories I and II were projected onto a transmission image of the cross-section of the cell of interest. (b) Fluorescence lifetime versus time for trajectories I and II shown in (a). (c) Traces I and II, as shown in (a), are plotted in 3D with pseudo color representing different fluorescence lifetimes. The color bar on the right indicates the fluorescence lifetime pseudo-color scale. (d) The fluorescence lifetime of the beads in different pH buffers, i.e., pH 4.56, pH 5.60, pH 7.05, and pH 7.77.

In order to find out whether the pH is the decisive factor for the lifetime changes, the lifetimes of the microspheres at four different pHs, i.e. pH 4.56, pH 5.60, pH 7.05, and pH 7.77, were measured. The exact pH was measured with a pH meter (S210, Mettler Toledo). As is shown in Figure 4(d), the four lifetime are 4.30 ± 0.10 ns, 4.35 ± 0.15 ns, 4.17 ± 0.09 ns, and 4.18 ± 0.13 ns, respective. Although the lifetime at acidic solutions is a bit longer than that at alkaline solutions, there are no obvious difference among the four lifetimes. So, the pH may contribute a little but there should be some other complex factors which caused the significant changes of fluorescence lifetime in the cell experiment.

In summary, we proposed a 4D-tracking method for observing dynamic events in living cells, providing dynamic trajectories in three dimensions and the impact of microenvironment during the dynamic events. By combining DH-PSF with fluorescence lifetime measurement, multiple particles within a depth range of several microns can be located and tracked simultaneously. Furthermore, their local environment, as reflected by dependent the fluorescence lifetime, can also be monitored. The method was numerically simulated and then demonstrated experimentally. The numerical simulation demonstrated that, based on reasonable settings, particles positioned within a depth of 4 µm can be located. Average localization precisions of 10, 15, and 33 nm in the x , y , and z directions, respectively, as well as a fluorescence lifetime precision of 40 ps, were obtained. The method was also implemented experimentally, by adapting a DH-PSF microscopy and using a streak camera, and then used to observe endocytosis in living RAW cells. It should be noted that the three-dimensional localization method adopted in this paper is the DH-PSF method, but other similar methods, such as MUM [41] and DDCM [22, 38], which has larger effective DOF, could also be used.

This investigation has established a reliable approach for the study of dynamic events in living cells, the method may reveal relationships between explicit trajectories of vesicles, proteins, or other biomolecules and the implicit microenvironment, providing a fresh perspective from which to gain understanding of the complex functions of cells. For example, for many cellular compartments which are not bound by membranes, it remained elusive how they concentrate molecules, maintain and regulate their structures, control their compositions and modulate internal biochemical activities. The method presented here may be useful in revealing relationship between the behavior of the moving vesicles/proteins and these membraneless compartments.

In addition, this study has also provided a methodology for designing and constructing FLIM systems with dynamic tracking for biomolecules. In the future, the study may be exploited by using some specific surface-functionalized beads with the strategy adopted in some papers published recently [42, 43]. We believe that this will be proven very beneficial for investigating the physiological and pathological processes of cell.
